# Anti-Typhoid Inoculation

**Published:** 1900-09-29

**Authors:** 


### ANTI-TYPHOID INOCULATION.

A long letter from Dr. Donald Ross, full of indignation at the anti-science attitude of the Government, appeared in last Monday's *Times*. In it he draws attention to the fact that in 1898 the Secretary of State for India refused to permit officers and soldiers to undergo inoculation against typhoid fever, even voluntarily, and he adds that this ruling was made in opposition to the advice and wish of the Government of India, and seems to be still in force. He says that he has called attention to this subject, not for the sake of finding fault, but in order to give a striking instance of the constant neglect and even opposition which the highest efforts of pathological science met with in this country. On the whole, we may, perhaps, be allowed to doubt whether the illustration quite bears out the text. It must be remembered that, in dealing with the army, it is impossible for the authorities to divest themselves of responsibility for any proceeding which they permit, and, taking this view of their responsibility, we are not surprised to find that the Government refused for a time in 1898 to sanction such a very experimental proceeding as inoculation for typhoid then was. Dr. Ross is a scientific man, and swears by his own gods. He is indignant that the Government should have declined to be convinced, and he can see no excuse for their hesitation, when "the Pasteur Institute, Haffkine, and Wright himself" were in favour of the process. Now, we have every respect for science—indeed, if not scientific, what are we?—but even judging after the event, and with all the added knowledge of two years, we doubt whether it can be held as a serious charge against the Government that, so far back as two years ago, they held their hand, and did not at once obey the mandate of science as expressed by "the Pasteur Institute, Haffkine, and Wright himself" in regard to anti-typhoid inoculation.

